# Dental age estimation using the Kvaal method—an evaluation of length and width ratios: a systematic review

**DOI:** 10.1007/s12024-023-00575-9

**Published:** 2023-02-11

**Authors:** S. Kazmi, Syed Jaffar Abbas Zaidi, Gowri Vijay Reesu, Simon Shepherd

**Affiliations:** 1 Department of Oral Biology, School of Dentistry, Shaheed Zulfiqar Ali Bhutto Medical University, Islamabad, Pakistan; 2 Department of Oral Biology, Dow Dental College, Dow University of Health Sciences, Karachi, Pakistan; 3 Saint John, New Brunswick, Canada; 4 Dundee Dental Hospital & Research School, University of Dundee, Park Place, Dundee, DD1 4HN UK

**Keywords:** Dental age estimation, Kvaal method, Tooth length and width ratios, Age correlation

## Abstract

This study aimed to systematically review the correlational accuracy between width ratios and length ratios based on the Kvaal methodology with chronological age. This systematic review followed Preferred Reporting Items for Systematic Reviews and Meta-Analysis (PRISMA). The search strategy included ProQuest, PubMed, Science Direct, and Taylor and Francis and Willey online without time or language restriction using Kvaal method of age estimation as key words for the search up to December 2021. A team of two researchers independently selected the studies and extracted the data. The Covidence platform was used to systematically organize all titles. The full texts of eligible studies were analyzed. Risk of bias (RoB) was assessed using a modified (to the specific characteristics of this systematic review) checklist based on Strengthening the Reporting of Observational Studies in Epidemiology (STROBE) statement checklist for observational studies. A total of 658 articles were initially reviewed, but 22 were selected for inclusion. The risk of bias was estimated to be unclear to low overall. Among the length ratios, ratio R showed a strong association with chronological age, followed by ratio P. For the width ratios, ratio B demonstrated a close association with chronological age, followed by ratio C. The results suggest that width ratios correlate better with chronological age than length ratios. This systematic review suggests the width ratios are more strongly associated with chronological age than the length ratios. Using a width ratio could serve as a convenient and rapid way to estimate dental age. Our results apply equally to all types of ethnic groups.

## Introduction

Dental age estimation (DAE) methods rely on tooth development stages and also on age-related changes in teeth [1, 2]. To assess age estimation in children, tooth development stages are commonly employed while post-formation changes within the tooth are applied in adults [1, 3, 4].

Dentin deposition is a well-recognized post-formation change associated with aging teeth which continues throughout life; as a result, pulp becomes narrow [4]. Therefore, dentine apposition can serve as a useful dental age predictor in adults [1, 3, 5].

The most appropriate DAE method for an adult depends on whether the subject is living or deceased. Methods based on secondary dentine apposition measurements at different levels and tooth sectioning are destructive, and therefore not suitable for living subjects [6, 7]. On the contrary, methods based on secondary dentine apposition and dental radiographs are easy, non-destructive, and applicable to living individuals. These non-invasive methods are mainly based on two-dimensional (2D) or three-dimensional (3D) images [1, 3, 5, 7]. With advancing age, secondary dentine apposition reduces pulp size, and so represents an important criterion for radiographic assessment methods [4, 8].

In 1995, Kvaal et al. provided a DAE method based on secondary dentine formation using 2D dental radiographs (Table 1). Since then, researchers have applied Kvaal’s method to investigate the correlation between pulp tooth ratios in different populations using (2D) and (3D) images [9–11]. In Kvaal’s original study, the correlation values ranged from 0.56 to 0.76 from a combination of length and width ratio regression equations. However, several studies showed that correlation values were higher with width ratios [12, 13].

Numerous research publications have applied the Kvaal age estimation method [10, 14–16]. Some studies have also applied modifications to this method using width ratios only [12, 13]. Results suggest that width ratios correlated better with chronological age than do length ratios. However, to the best of our knowledge, no review has assessed the potential correlation between length and width ratios with age. This review aimed to summarizes the published correlation results between length and width ratios according to the Kvaal method. Our study will help to achieve a better understanding of the correlation between length and width ratios and chronological age.

## Material and methods

This systematic review was conducted in seven phases: These seven phases included study protocol and registration, search strategy, inclusion criteria, exclusion criteria, reviewing process and studies selection, data extraction and management, and quality assessment and risk of bias. No formal ethics approval was required for this review, as it did not involve human subjects and was based solely on the analysis of published studies. Based on the “PECOS” framework (population, exposure, comparison, outcome, type of study), the review question was as follows “Are the accuracy of width ratios better correlated with age than length ratios?”.

### Study protocol and registration

**Fig. 1:**
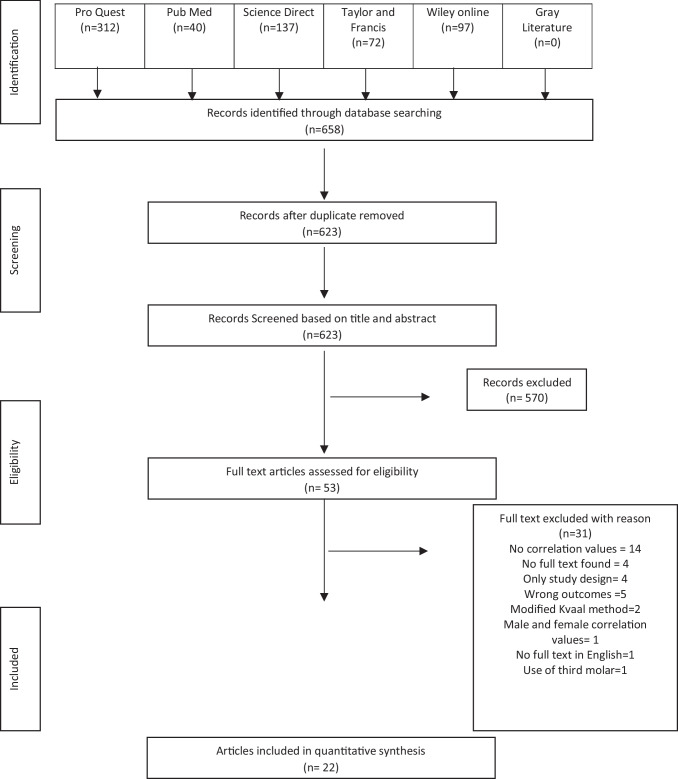
Flow chart of the literature search and selection criteria

This study followed the Guidelines of Preferred Reporting Items for Systematic Reviews and Meta-Analysis (PRISMA) [17] (Fig. 1). As the protocol is designed exclusively for forensic sciences, it could not be registered with PROSPERO’s International Prospective Register of Systematic Reviews (PROSPERO).

### Search strategy

Five electronic databases (ProQuest, PubMed, Science Direct, Taylor & Francis and Wiley Online) were searched using the following keywords: *Kvaal method of age estimation* OR *Kvaal dental age estimation* [18]. No language restrictions were imposed. The literature search included papers published after the original manuscript was published by the authors up to December 2021. In addition, grey literature was searched using IADR data base.

### Inclusion criteria

Included in the analysis were original studies reporting the use of the Kvaal method of age estimation and also detailing the correlation between chronological age and pulp-tooth ratios according to the Kvaal method.

### Exclusion criteria

The exclusion criteria included research whereby the Kvaal method was applied to developing teeth or where the sample included individuals younger than 12 years of age, as post-formational changes could not be taken into account. It can be argued that stage development methods that include tooth development atlases are better suited to these populations. Furthermore, any studies reporting modification to the Kvaal methodology, studies with fewer than 20 subjects, case series, case reports, letters to the editor, and book chapters were also excluded.

### Reviewing process and studies selection

A total of 658 papers were uploaded to EndNote for collection based on titles and abstracts. The search results were transferred to the Covidence online platform (https://community.cochrane.org/help/tools-and-software/covidence/ Accessed 02 December 2021) for screening and categorization, since this Covidence software facilitates the screening process with a clear audit trail.

Initially, duplicate references were excluded. Each review process step was conducted independently by two reviewers (SK and JZ). Abstracts and titles were independently screened based on the criteria listed, and reviewers considered and compared their selections to reach a consensus. Abstracts and full-text articles were independently evaluated for eligibility. In any discrepancies, a third reviewer (SM) was consulted. The PRISMA flow chart shows the process and results of the literature search and study selection (Fig. 1).

### Data extraction and management

An organized assessment of systematic review articles’ titles and abstracts was performed using a primary screening and data extraction tool called Evidence (Covidence). The collected information was organized in an Excel spreadsheet under the headings of: author, country, year, and the best correlation between tooth length, width, and age (Table 2).

**Table 1:** Measurements of length and width with notation and description

Notation	Ratios with descriptions
P	Ratio between length of pulp and root
T	Ratio between length of tooth and root
R	Ratio between length of pulp and tooth
A	Ratio between width of pulp and root at enamel-cementum junction (level A)
B	Ratio between width of pulp and root at mid-point between level C and A (level B)
C	Ratio between width of pulp and root at mid-root level (level C)

**Table 2:** Best correlation values between teeth (FDI) and Kvaal dental ratios with age

Tooth numeration FDI (Federation Dentaire International)											
Author	Country	11/21	12/22	15/25	32/42	33/43	34/44	13/23	3 Max teeth	3 Man teeth	6 teeth
Kvaal et al. [1]	Norway	P	B	A	A	A	A	-	-	-	-
Kolltveit et al. [34]	Caucasian	R	B, C	B	R	R	A	-	-	-	-
Bosmans et al. [21]	Belgium	A	A	A	A	B	A	-	-	-	-
Landa et al. [10]	Caucasian	-	-	-	R	C	B	-	-	-	-
Sharma and Srivastava [25]	India	C	C	C	C	P	P	-	-	-	-
Saxena et al. [24]	India	-	-	-	-	-	-	C	-	-	-
Erbudak et al. [14]	Turkey	R	B	R	P	A, B	B	-	-	-	P
Limdiwala and Shah [22]	India	-	-	-	-	-	-	-	A	B	B
Misirlioglu et al. [26]	Turkey	-	-	-	B	C	C	C	-	-	-
Patil et al. [15]	India	B, C	-	-	-	-	-	-	-	-	-
Karkhanis et al. [16]	Australia	C	C	A	A	P	A	-	-	-	-
Mittal et al. [23]	India	A	C	C	A	C	B	-	-	-	-
Marroquin Penaloza et al. [31]	Malaysia	A	B	-	-	-	-	B	-	-	-
Marroquin Penaloza et al. [30]	Australian	B	C	B	B	C	C	-	-	-	-
Roh et al. [12]	Korean	B	C	B	B	T, A	B	-	B	ABC	B
Li et al. [9]	China	B	B	R	P	A, B	B	-	B	B	B
Akay et al. [11]	Turkey	B	-	-	-	-	-	-	-	-	-
Hisham et al. [28]	Malaysia	B	B, C	P	C	C	C	-	-	-	-
Hisham et al. [33]	Malaysia	A, B	C	B	B	B	B	-	-	-	-
Li et al. (2020)	China	-	-	-	-	R	-	B	-	-	-
Chandan et al. [27]	India	A	A	B	A	B	A	-	-	-	-
Vossoughi et al. [32]	Iran								A	A	B

P ratio between length of pulp and root, T ratio between length of tooth and root, R ratio between length of pulp and tooth, A ratio between width of pulp and root at CEJ (level A), B ratio between width of pulp and root at midpoint between level C and A (level B), C ratio between width of pulp and root at mid-root the level (level C)

### Quality assessment and risk of bias

Reviewers (SK and JZ) independently assessed the studies using a checklist based on the Strengthening the Reporting of Observational Studies in Epidemiology (STROBE) statement checklist for cross-sectional studies [19]. The six domains included title and abstract, introduction, methods, results, discussion, and other information. The checklist was modified to better encompass the specific characteristic of the articles included in this systematic review (Appendix).

A three-part scoring system was applied for each item. If the criterion was not met, a value of 0 was awarded. In the case of partial fulfillment of the necessary criteria, 1 was awarded. In the case of complete fulfillment, a value of 2 was awarded. A study was considered under these criteria as low value if the overall score was below 22, of an average value if scored between 23 and 29 and high value if the score was above or equal to 30. The final score was determined by the average of two reviewers’ scores [20].

## Results

### Study selection

The initial search of the six electronic databases identified 658 studies based on the inclusion and exclusion criteria. After removing duplicates, 623 titles and abstracts were selected for further reading. Of these, 53 were subsequently selected for full-text review to assess their eligibility according to the criteria. Finally, 22 studies satisfied the criteria and were selected for inclusion in the systematic review (Fig. 1).

### Characteristics and quality of the studies included

As a result of the search strategy performed, 658 different articles were identified. Of these, 22 were included in the systematic review. Most studies included were from Indian and Caucasian populations [10, 14, 15, 21–27], with limited studies from other populations.

The included studies used different radiographic images. Most of them considered orthopantomogram (OPG) [1, 9, 10, 12, 14–16, 21–24, 28–30]. Two used periapical radiographs and cone beam computed tomography (CBCT) images [15, 25, 31, 32]. One used a combination of CBCT and multi-detector row computed tomography (MDCT) and one used CBCT images of extracted teeth for measurements [11, 33].

Regarding teeth selected, for correlation between chronological age and Kvaal length and width ratios, most of the included studies used the six teeth as recommended in the original Kvaal method [1, 10, 16, 23, 24, 27, 28, 31, 33, 34]. Four studies used a combination of the Kvaal recommended six teeth with three mandibular and three maxillary teeth and a sum of six teeth [9, 12, 14, 21]. Two studies used canines [24, 29].Two studies used a combination of three mandibular and three maxillary teeth and the sum of mandibular and maxillary teeth [22, 32]. One study used a combination of three mandibular teeth and three maxillary teeth [10]. One study used a combination of three mandibular teeth and three maxillary teeth [26]. One study used the maxillary canine, and one study used six teeth with mandibular central incisor and second premolar [11].

### Quality assessment

Quality assessment scores were between 25 and 32.75. Overall, no study was of low quality, 12 were of average quality, and 10 were of high quality. The score of each study has been included in the Appendix.

### Correlation results

This systematic review comprehensively explored 22 articles using the Kvaal method of DAE published in 11 countries. The Kvaal method requires odontometric analysis of six different single-rooted teeth. The observer employing this method needs to perform nine measurements per tooth and subsequently calculate several ratios.

Table 2 shows the corresponding maximum correlation values between chronological age and the Kvaal length and width ratios and teeth. For the width ratios, the highest correlation value belonged to value B, followed by A and C, respectively. Considering the length ratios, value P had a greater correlation than R and T. Generally, width ratios B, A and C had the highest correlation values.

Considering the application of the Kvaal methodology to different radiographic images, we found that width ratios exhibited higher correlation values than the length ratios. Studies using OPG radiographs revealed that ratios A and B showed a relatively high correlation with age, followed by the ratio C, and studies using periapical radiographs concluded that ratio C had a high correlation with age followed by ratio B. The same trend of results was detected in the 3-d studies indicating that ratio B strongly correlated with age. Moreover, studies using OPG and periapical radiographs found that the best correlation was observed for ratio C.

Regarding length, width ratios with teeth, and age, width ratios are more closely correlated with age. Ratio B showed a relatively strong correlation with age in the maxillary central incisor and the maxillary second premolar, while ratio C showed a high correlation with age in the maxillary lateral incisor and mandibular canines. The best correlation ratio A was seen in applying the method to the mandibular lateral incisor. Additionally, we found that ratios B and C showed strong correlations in both the mandibular canine and the maxillary canine. Based on the results of this study, it is obvious that all the width ratios demonstrated a better correlation with age than did length ratios.

## Discussion

There is a relative dearth of literature analyzing the intricacy of the pattern of secondary dentine formation and pulp chamber constriction. Interpretations regarding the relationship between secondary dentine formation and specific regions within a tooth have been conflicting. Philippas reported that the site of secondary dentine formation is more in the floor of the pulp chamber than in the roof with age [35], whereas Murray et al. also reported asymmetrical amounts of secondary dentine in the crown and root, but with more in the root [36]. However, Oi et al. reported no difference between secondary dentine formation and specific regions within a tooth with advancing age [37].

While there are differences in the location of dentine formation, the present study suggests that width ratios are more correlated with age than length ratios. Ratio B (ratio between the width of pulp and root at the midpoint between levels C and A) showed the strongest relationship with age. This finding is supported by other authors who have found similar high associations between Ratio B with age [12, 14].

Several studies have demonstrated that pulp width is a better indicator of age than pulp length based on pulp tooth volume ratios. Using microCT scans of mandibular premolar teeth, Aboshi et al. compared the pulp tooth volume ratio at four different levels and found highest association in the coronal one-third of the root [38]. Paewinsky et al. evaluated the digital OPGs of a German population using Kvaal et al.’s approach and width ratios alone provided higher correlations and increased accuracy and best associations with age were determined to be at level A. In terms of teeth, mandibular first premolars, level B showed the best correlation with age [13]. Similarly, some authors who have used lengths and width measurements to calculate age have proposed that the use of width parameters without the length ratios achieves higher accuracy of results [12, 13, 26, 29].

Several studies have evaluated the accuracy of the Kvaal method using 2-D radiographs, namely OPGs, which can acquire images of six teeth in a single image. Taking periapical radiographs seems cumbersome and complex, so most studies used OPGs to determine the applicability of the Kvaal method, but their results invite conflict. OPG radiographs have been shown to be inferior to periapical radiographs secondary to the inferior image quality and unequal magnification. Furthermore, Adarsh et al. pointed out that 3-D radiographic techniques are the most accurate for undertaking measurements of teeth [39].

In our sample, studies based on 2-D and 3-D images were included, and we found that width ratios showed a better correlation with chronological age than length ratios. Therefore, we suggest using width ratios in age estimation regardless of whether 2-D or 3-D images, to achieve higher accuracy.

When reviewing the literature, we found that incorporating Kvaal’s suggested length and width ratios into linear regression equations for age estimation yielded varied results from 30 to 10 years. Paewinsky used only width ratios according to Kvaal for age estimation and found that the estimated age and chronological age differed by 6.4 years [13]. Similarly, Roh et al. used width ratios in their regression model, and the results revealed a narrow gap between estimated and chronological age [12].

Bang G, in 1989, highlighted that secondary dentine formation is affected by genetic and environmental factors, thus causing differences in age estimation [40]. It is acknowledged that prediction accuracy can be influenced by many mitigating factors such as differences in tooth development, genetic background, tooth size, bite-force magnitude, variations in the tooth dimensions, and pulp shape within global populations and within given specific populations [41–45].

Another important reason for the differences in the results of studies using the Kvaal method may be the reproducibility and accuracy of morphometric measurements. Schulze R et al. found that vertical measurements were less reproducible and accurate than horizontal measurements and that root width measurements have greater reproducibility than pulp width measurements [46]. The vertical measurements may appear to be influenced by chewing habits, tooth wear, bruxism, attrition, and dietary patterns, thus inducing secondary dentine formation at the roof of the pulp chamber [40, 44].

This systematic review of 22 studies from 10 different global populations strongly suggests that width ratios are preferable to length ratios. The results suggest that despite these factors and the population specificity effect, the effects of these factors can be minimized by using width ratios in age estimation formulas. Value B, the measurement of width at the midpoint between the CEJ and mid-root, seems to be the best age predictor.

In our view, one major limitation of this review is the fact that the search strategy was based on a minimal subset of specific keywords describing the Kvaal method of age estimation. The possibility that additional articles could have been identified by adding other terms cannot be excluded. There is scope to widen the search to include relevant studies indexed in grey literature databases. Another limitation the authors feel important to highlight is that no meta-analysis could be performed in the present study, as insufficient homogenous data were available.

## Conclusion

According to the original Kvaal method, researchers needed to perform six specific measurements per radiographic image of a tooth and then calculate various ratios to arrive at an accurate age estimation. This review suggests and provisionally concludes that width ratios alone may be used to provide an alternative age estimation model with acceptable accuracy. In the published studies using the Kvaal method, width ratios correlate better with chronological age than length ratios. Ratio B showed the strongest correlation with age. Considering an age estimation in the adult population, an age assessment might focus more on width than length variables. The pulp tooth width ratio at level B alone or combined with other ratios might also help estimate chronological age.

## Key points

1. Kvaal age estimation methodology explored to understand variables which correlate best with age.
2. The original method combines radiographic length and width ratios using tooth:pulp area.
3. The results suggest that width ratios are better correlated with age than are length ratios.
4. A smaller subset of measures may improve ease and maintain accuracy in age estimation.

## Data Availability

The data used to support the findings of this study are available from the corresponding author upon request.

## Appendix

**Table:**

**Questions**	**Check list modified based on STROBE**
1	Title or abstract must contain information about the Kvaal method used to assess the dental age
2	Background and reason for study outlined
3	Aims and objectives of the study mentioned
4	Clear presence of study design and methodology
5	Data collection details
6	Inclusion details of the subjects
7	Details of the teeth included in the study
8	Description of the Kvaal method
9	Sufficient detail about the statistical method used
10	Age distribution of the sample
11	Demographic or characteristics of the subjects
12	Correlation results between teeth and Kvaal dental ratios
13	Overall results detailing dental age estimation
14	Results with reference to study objectives
15	Limitations of the study
16	Overall interpretations of results and comparison with similar studies
17	Findings of study applicability to other studies
18	Funding details

**Table:**

**Quality Assessment Strobe Score Reviewer 1**																			
**Studies**	**1**	**2**	**3**	**4**	**5**	**6**	**7**	**8**	**9**	**10**	**11**	**12**	**13**	**14**	**15**	**16**	**17**	**18**	**Total**
Kvaal et al. [1]	2	2	2	2	2	2	2	2	2	2	2	2	2	2	1	1	2	0	23
Kolltveit et al. [34]	1	1	2	2	2	0	2	2	1	2	2	2	0	2	0.5	1	2	0	25
Bosmans et al. [21]	2	2	2	1	2	1	2	0	2	2	2	2	2	2	0	0.5	2	0	28.5
Landa et al. [10]	2	2	2	2	2	2	2	2	2	2	2	2	2	1	0.5	1	2	0	30.5
Sharma and Srivastava [25]	2	1	0	1	0	2	2	2	2	0	0	2	2	1	0	1	1	0	21
Saxena et al. [24]	2	1	2	1	2	2	2	1	0	2	1	2	2	2	0	0.5	1	0	23.5
Erbudak et al. [14]	2	2	2	2	2	2	2	2	2	2	2	2	2	2	2	1	2	0	33
Limdiwala and Shah [22]	2	1	2	1	2	2	2	2	2	0	0	2	2	2	1	1	1	0	25
Misirlioglu et al. [26]	2	2	2	2	2	2	2	2	2	2	2	2	2	2	0	2	2	2	34
Patil et al. [15]	2	1	2	1	1	0	2	1	1	0	2	2	1	2	0	0.5	1	0	19.5
Karkhanis et al. [16]	2	2	2	2	2	2	2	2	2	0	2	2	2	2	0	2	2	2	32
Mittal et al. [23]	2	2	2	2	2	2	2	2	2	2	0	2	2	2	0	0.5	1	0	27.5
Marroquin Penaloza et al. [31]	1	1	2	2	2	2	2	2	2	0	2	2	2	2	0	1	2	2	29
Marroquin Penaloza et al. [30]	2	2	2	1	2	0.5	2	2	2	2	2	2	2	0.5	2	1	1	2	30
Roh et al. [12]	2	2	2	1	1	1	2	2	2	0	2	2	2	2	1	2	2	0	28
Li et al. [9]	2	2	2	1	2	2	2	2	2	2	2	2	2	2	0	2	2	2	33
Akay et al. [11]	0	1	2	1	2	2	2	2	2	2	2	2	2	2	0.5	1	1	2	28.5
Hisham et al. [28]	2	2	2	2	2	1	2	2	2	2	2	2	2	2	1	1	0	2	31
Hisham et al. [33]	1	1	2	2	2	2	2	1	2	0	2	2	1	2	0	2	2	0	26
Li et al. [29]	2	0.5	2	1	2	2	2	2	2	0	2	2	2	1	0	1	1	2	26.5
Chandan et al. [27]	2	2	1	1	2	1	2	2	2	2	2	2	2	2	0	1	2	2	30
Vossoughi et al. [32]	2	1	2	1	2	2	2	1	0	2	1	2	2	2	0	0.5	1	0	33

**Table:**

**Quality Assessment Strobe Score Reviewer 2**																			
**Studies**	**1**	**2**	**3**	**4**	**5**	**6**	**7**	**8**	**9**	**10**	**11**	**12**	**13**	**14**	**15**	**16**	**17**	**18**	**Total**
Kvaal et al. [1]	2	2	2	1	2	2	2	2	2	2	2	2	2	2	0	0.5	2	0	29.5
Kolltveit et al. [34]	0.5	1	2	2	2	2	2	2	1	2	2	2	0	2	0	1	2	0	25.5
Bosmans et al. [21]	2	2	2	1	2	2	2	0	2	2	2	2	2	2	0	0.5	2	0	27.5
Landa et al. [10]	2	1	2	1	2	2	2	2	2	2	2	2	2	2	0.5	1	2	0	29.5
Sharma and Srivastava [25]	2	0.5	0	1	0	2	2	2	2	0	0	2	2	0.5	0	0.5	2	0	18.5
Saxena et al. [24]	2	1	2	1	2	2	2	2	2	0	2	2	2	1	0	0.5	1	0	24.5
Erbudak et al. [14]	2	2	2	2	2	2	2	2	2	2	2	2	2	2	2	1	2	0	31
Limdiwala and Shah [22]	2	1	2	1	2	2	2	0	2	0	0	2	2	1	1	1	0.5	0	21.5
Misirlioglu et al. [26]	2	2	2	2	2	2	2	2	2	2	2	2	2	2	0	2	2	0	32
Patil et al. [15]	2	0.5	2	1	2	2	2	2	2	0	2	2	2	2	0	0	2	0	25.5
Karkhanis et al. [16]	2	2	2	2	2	2	2	0	2	0	2	2	2	2	0	2	2	0	26
Mittal et al. [23]	2	1	2	1	2	2	2	0	2	2	1	2	2	2	0	0.5	1	0	24.5
Marroquin Penaloza et al. [31]	2	2	2	2	2	2	2	2	2	0	2	2	2	2	0	1	2	0	29
Marroquin Penaloza et al. [30]	2	1	2	2	2	2	2	1	2	2	2	2	2	2	0	0	2	2	30
Roh et al. [12]	2	2	2	2	0	2	2	2	2	0	0.5	2	2	2	0	2	2	0	26.5
Li et al. [9]	2	1	2	2	2	2	2	2	2	2	2	2	2	2	0.5	1	2	2	32.5
Akay et al. [11]	0	2	2	2	2	2	2	2	2	2	2	2	2	2	0	1	2	2	31
Hisham et al. [28]	2	1	2	2	2	2	2	2	2	2	2	2	2	2	0	1	2	0	30
Hisham et al. [33]	2	0.5	2	1	2	2	2	0	2	0	2	2	2	2	0	1	2	0	24.5
Li et al. [29]	2	1	2	2	2	2	2	2	2	0	2	2	2	2	0	0.5	2	2	29.5
Chandan et al. [27]	2	2	1	2	2	2	2	2	2	2	2	2	2	2	0	0.5	2	2	31.5
Vossoughi et al. [32]	2	2	2	2	2	2	2	2	2	2	2	2	2	2	0	0.5	2	2	32.5

**Table:**

**Sum of scores of Reviewers 1 and 2**																				
**Studies**	**1**	**2**	**3**	**4**	**5**	**6**	**7**	**8**	**9**	**10**	**11**	**12**	**13**	**14**	**15**	**16**	**17**	**18**	**Total**	**Total/2**
Kvaal et al. [1]	4	4	4	3	4	4	4	4	4	4	4	4	4	4	1	1.5	4	0	61.5	30.75
Kolltveit et al. [34]	1.5	2	4	4	4	2	4	4	2	4	4	4	0	4	0.5	2	4	0	50	25
Bosmans et al. [21]	4	4	4	2	4	3	4	0	4	4	4	4	4	4	0	1	4	0	54	27
Landa et al. [10]	4	3	4	3	4	4	4	4	4	4	4	4	4	3	1	2	4	0	60	30
Sharma and Srivastava [25]	4	1.5	0	2	0	4	4	4	4	0	0	4	4	1.5	0	1.5	3	0	37.5	18.75
Saxena et al. [24]	4	2	4	2	4	4	4	3	2	2	3	4	4	3	0	1	2	0	48	24
Erbudak et al. [14]	4	4	4	4	4	4	4	4	4	4	4	4	4	4	4	2	4	0	66	33
Limdiwala and Shah [22]	4	2	4	2	4	4	4	2	4	0	0	4	4	3	2	2	1.5	0	46.5	23.25
Misirlioglu et al. [26]	4	4	4	4	4	4	4	4	4	4	4	4	4	4	0	4	4	2	66	33
Patil et al. [15]	4	1.5	4	2	3	2	4	3	3	0	4	4	3	4	0	0.5	3	0	45	22.5
Karkhanis et al. [16]	4	4	4	4	4	4	4	2	4	0	4	4	4	4	0	4	4	2	60	30
Mittal et al. [23]	4	3	4	3	4	4	4	2	4	4	1	4	4	4	0	1	2	0	52	26
Marroquin Penaloza et al. [31]	3	3	4	4	4	4	4	4	4	0	4	4	4	4	0	2	4	2	58	29
Marroquin Penaloza et al. [30]	4	3	4	3	4	2.5	4	3	4	4	4	4	4	2.5	2	1	3	4	60	30
Roh et al. [12]	4	4	4	3	1	3	4	4	4	0	2.5	4	4	4	1	4	4	0	54.5	27.27
Li et al. [9]	4	3	4	3	4	4	4	4	4	4	4	4	4	4	0.5	3	4	4	65.5	32.75
Akay et al. [11]	0	3	4	3	4	4	4	4	4	4	4	4	4	4	0.5	2	3	4	59.5	29.75
Hisham et al. [28]	4	3	4	4	4	3	4	4	4	4	4	4	4	4	1	2	2	2	61	30.5
Hisham et al. [33]	3	1.5	4	3	4	4	4	1	4	0	4	4	3	4	0	3	4	0	50.5	25.25
Li et al. [29]	4	1.5	4	3	4	4	4	4	4	0	4	4	4	3	0	1.5	3	4	56	28
Chandan et al. [27]	4	4	2	3	4	3	4	4	4	4	4	4	4	4	0	1.5	4	4	61.5	30.75
Vossoughi et al. [32]	4	4	4	4	4	3	4	4	4	4	4	4	4	4	0	2.5	4	4	65.5	32.75
